# The effects of Leventhal’s self-regulation model-based educational intervention on stress, anxiety, and depression in women with multiple sclerosis: a randomized educational trial

**DOI:** 10.3389/fneur.2025.1521112

**Published:** 2025-05-09

**Authors:** Mahsa Hosseini, Soheila Shamsikhani, Ali Jadidi, Fatemeh Rafiei

**Affiliations:** ^1^Shazand School of Nursing, Arak University of Medical Sciences, Arak, Iran; ^2^Department of Nursing, School of Nursing and Midwifery, Arak University of Medical Sciences, Arak, Iran; ^3^Department of Epidemiology and Biostatistics, School of Public Health, Tehran University of Medical Sciences, Tehran, Iran

**Keywords:** multiple sclerosis, Leventhal’s self-regulation, anxiety, stress, depression

## Abstract

**Background:**

Multiple sclerosis (MS) is a long-term, progressive neurological condition that affects the myelin sheath of nerve cells in the central nervous system. Mental health concerns are often overlooked in individuals with MS, stemming from various aspects of the illness, such as its inherent characteristics and symptoms. The present research aimed to examine the impact of Leventhal self-regulation training on stress, anxiety, and depression in MS patients.

**Methods:**

The current study was conducted as a randomized educational trial of 60 women referred to the MS Association in Arak City in 2024. The study implemented self-regulation training via the Leventhal model, which consisted of an initial face-to-face session to provide basic knowledge, eight offline sessions, and two online group sessions. The data were input into SPSS 26 software. Chi-square tests and Fisher’s exact tests were utilized, independent t-tests, and Mann–Whitney’s nonparametric equivalent. A repeated measures analysis of variance was conducted to assess the changes over three time points. Additionally, the Bonferroni *post hoc* correction was utilized. In the present study, data collection consisted of two parts: the demographic information form and the Depression Anxiety Stress Scale (DASS) questionnaire. The Iranian registry Clinical Trial code (IRCT) is IRCT20220703055351N3.

**Result:**

The findings of this research indicated that there were no disparities in anxiety (*p* = 0.072), stress (*p* = 0.067), or depression (*p* = 0.170) between the control and experimental groups before the intervention. The mean (± standard deviation) anxiety, stress, and depression scores of the experimental group changed from 4.89 (4.34), 8.55 (5.57), and 6.82 (4.26) to 1.96 (2.48), 4.93 (4.55), and 4.37 (3.73) after the intervention, respectively (*p* < 0.05).

**Discussion:**

Recent research has shown that training based on the Leventhal model is effective in reducing stress, anxiety, and depression in MS patients. Additionally, the results showed that the ability of Leventhal training to improve depression was stable for one month.

**Clinical trial registration:**

https://irct.behdasht.gov.ir/, IRCT20220703055351N3.

## 1 Introduction

Multiple sclerosis (MS) is a chronic, degenerative neurological condition characterized by damage to the myelin sheath within the central nervous system (1). In 2020, the global incidence of MS was reported at 35.9 cases per 100,000 individuals (2). In Iran, approximately 100 out of every 100,000 people are diagnosed with MS, a figure that continues to rise (3). Notably, around 75% of MS patients are female (4), resulting in a higher prevalence of the disease among women in Iran compared to global averages (5).

A significant concern in the management of MS is the often-overlooked mental health of those affected by the condition. The impact of MS on mental health is complex and multifaceted, typically arising from the disease’s characteristics and its associated symptoms (6). Psychological symptoms are frequently observed in individuals with MS; however, these symptoms often receive insufficient evaluation (7). Additionally, cognitive impairments are common among MS patients (8). Mental disorders, excluding schizophrenia, are more prevalent in MS patients than in the general population (9). The occurrence of mental disorders, such as anxiety and depression, is notably high among MS patients, and these conditions are critical to consider due to their influence on treatment adherence and overall quality of life (10). In fact, many individuals with MS experience the adverse effects of the disease, including anxiety, stress, and depression (11). Research indicates that comorbidities, such as depression, anxiety, cerebrovascular and cardiovascular diseases, and autoimmune disorders like diabetes, are associated with MS. These comorbid conditions can exacerbate MS symptoms, affect treatment adherence, and influence therapeutic outcomes (12). Consequently, anxiety, stress, and depression are regarded as both repercussions of MS and comorbidities.

The prevalence of anxiety and depression among MS patients is reported at 35.19 and 27.01%, respectively (13). The overall prevalence of these mental health issues among Iranian individuals with MS is 47% (14). Moreover, individuals with MS often experience heightened stress levels, which are believed to contribute to disease progression (15). In patients with autoimmune diseases such as MS, increased production of proinflammatory cytokines is linked to depression (16). Depression is a significant determinant of quality of life in MS patients and can even lead to suicidal ideation (17, 18). Furthermore, anxiety and depression, as emotional responses, can influence adaptation to the disease and affect patients’ functional capabilities (19). Therefore, prioritizing mental health aspects such as stress, anxiety, and depression in MS patients is essential.

In summary, MS can lead to various psychological consequences, including anxiety, stress, and depression, which can adversely affect multiple facets of health and overall well-being. One effective strategy for managing the physical and psychological complications associated with chronic diseases, including MS, is to enhance awareness and understanding of the disease.

A diminished perception of diseases is associated with an increase in negative outcomes, including stress, anxiety, and depression (20). Numerous research initiatives have been undertaken to enhance the understanding of various diseases, leading to improved management of their associated complications. These initiatives encompass peer support groups (21), religious psychotherapy methods (22), psychological training for caregivers (23), mindfulness-based cognitive therapy (24), pet therapy (25), and other interventions. However, these methods primarily focus on the cognitive dimension of illness perception. In contrast, Leventhal’s self-regulation theory emphasizes the importance of both cognitive and emotional factors in enhancing illness perception.

Introduced by Leventhal et al. in 1980, the self-regulation model illustrates the connection between individuals’ responses to perceived health threats and their subsequent actions (26). This model adapts to the context of disease and serves as an educational framework for understanding illness (27). Within this model, illness perception comprises two active parallel processes: 1. Recognition and objective interpretation of the threat; and 2. The interaction between knowledge and feelings (28). Each of these parallel processes consists of three stages (29): 1. Representation; 2. Coping; 3. Appraisal.

Illness perception significantly influences quality of life and can affect treatment adherence and psychosocial responses. Furthermore, the perception of a disease can impact its symptoms and consequences, such as stress, anxiety, and depression (30). According to this model, when patients adhere to the guidance and training provided to manage their condition, they develop a more accurate understanding of their illness, which in turn reduces the adverse consequences and mortality associated with the disease (31). It is anticipated that enhancing illness perceptions among individuals with multiple sclerosis will mitigate the negative consequences of the condition, such as stress, anxiety, and depression. Consequently, this study aims to evaluate whether an intervention based on Leventhal’s model can effectively reduce stress, anxiety, and depression in women with multiple sclerosis.

## 2 Methods

### 2.1 Study type and design

The current study was conducted as a randomized educational trial involving women referred to the MS Association in Arak City in 2024. For this study, the Iranian Registry Clinical Trial code (IRCT) is IRCT20220703055351N3.

### 2.2 Participant selection

The inclusion criteria specified that participants must be women aged between 18 and 50, possess access to a smartphone, be literate, and demonstrate communication skills in reading, writing, speaking, and listening. Participants must have had at least one year since the initial onset of the disease. Additionally, their medical history should be free of any other neurological, mental, or autoimmune disorders, and they must not be experiencing any uncontrolled acute illnesses during their participation in the study. Furthermore, their condition should not have relapsed, and they must not be using any narcotic substances. Participants are required to achieve a maximum score of 5.5 on the expanded disability status scale and should not have undergone any training that aligns with the objectives of this research. Informed consent was obtained from all participants.

The exclusion criteria included reluctance to maintain collaboration, disease recurrence, hospitalization for illness or other reasons, or significant life changes such as relocation, unexpected illness, or pregnancy.

### 2.3 Sample size calculation

The sample size was determined based on information from previous studies (32). With *α* set at 5% and *β* at 0.1, the following formula was applied:


$$n=\frac{{\left(z_{1-\frac{\alpha }{2}}+z_{1-\beta }\right)}^{2}\;({s_{1}}^{2}+{s_{2}}^{2})}{{\left(\mu_{1}-\mu_{2}\right)}^{2}}$$

The initial calculation indicated that 29 participants were required in both the control and experimental groups. To account for potential sample loss (10%), this number was increased to 32 participants per group, resulting in a total sample size of 64. During the research, four individuals were excluded, leaving a total of 60 participants. A convenience sampling method was employed. The Sealedenvelope website was utilized to generate a block random allocation list (33). Randomization was concealed through centralized randomization. Following participant enrollment, the researcher informed participants via SMS and consulted with another researcher to determine the random allocation of participants (34).

### 2.4 Intervention

This research introduced self-regulation training based on the Leventhal model. It comprised one in-person session for foundational knowledge, eight offline sessions for cognitive and emotional development, and two online group sessions for illness adaptation within one month. Virtual education included both synchronous (online) and asynchronous (offline) programs, with synchronous sessions held in groups and asynchronous sessions conducted individually.

The offline sessions incorporated multimedia elements, including podcasts, videos, and slides, which were uploaded to cloud storage (Dropbox). Links to each session were distributed via a Telegram group. The multimedia content addressed both the physical and emotional aspects of the disease. In collaboration with experts from various disciplines relevant to the disease’s dimensions, the materials were meticulously crafted based on articles, books, and expert insights (Table 1). The training was organized according to reliable scientific sources (1, 19, 30, 35–54).

**Table 1:** Explanation of intervention sessions.

Session	Week of sessions	Delivery format (Online/Offline)	Dimension of SMR	Topic	Description of the training
1	The first session aimed to enhance participants’ understanding of the research, conduct a survey for selecting appropriate platforms, and provide training on utilizing educational content				
2	First week	Offline	Cognitive	Identity	Introduction of the disease, mechanism of development, and incidence statistics
3		Offline	Emotional	Stress management	Mechanism of stress, identification of stressors, relationship between stress and MS
4	Second week	Offline	Cognitive	Cause	Explanation of the cause of the disease such as genetics, environmental factors, nutrition, lifestyle
5		Offline	Emotional	Problem-solving	Identifying the problem, generating solutions, evaluating solutions, prioritizing and choosing
6		Offline	Peer groups	To exchange the experiences of the participants, group discussion (with their peers)	
7	The third week	Offline	Cognitive	Timeline	Introduction to the types of MS. Relapse and remission periods. Progression of the disease
8		Offline	Emotional	Anger control	Identifying situations that cause anger, anger management techniques: leaving the place and
9	Fourth week	Offline	Cognitive	Controllability and consequences	Consequences, signs and complications, how to control and reduce complications
10		Offline	Emotional	Depression management	The mechanism of depression, identification of aggravating factors of depression, and the relationship between depression and MS
11		Offline	Peer groups	To exchange the experiences of the participants, group discussion (with their peers)	

The online group sessions were structured as peer groups to facilitate participant experience exchange. Participants were invited to engage in discussions with their peers and to create dynamic interactions with the researcher. Online sessions were conducted in a Telegram group.

**Figure 1:**
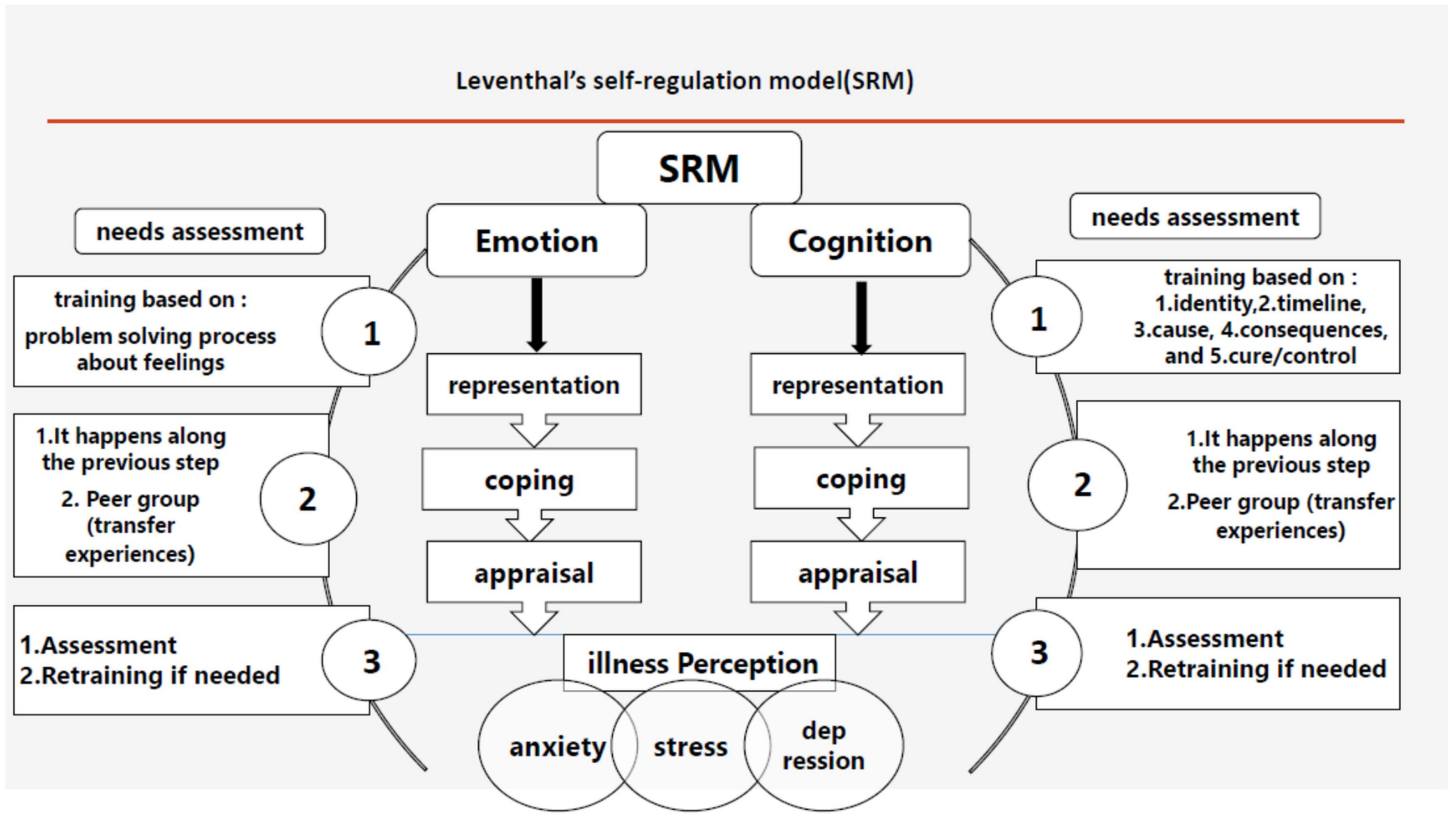
Leventhal’s self-regulation model.

The training was structured around the three stages of Leventhal’s self-regulation model: 1. Representation, 2. Coping, and 3. Appraisal (Figure 1). It was designed to encompass both cognitive and emotional dimensions.

#### 2.4.1 Representation

The objective is to develop a representation that effectively captures both cognitive and emotional dimensions. Within the cognitive domain, training encompasses five key dimensions: identity, cause, timeline, controllability, and consequences. This training was delivered to the experimental group via a multimedia link through Telegram every Monday. Given the interrelation between controllability and consequences regarding the disease, these two dimensions were consolidated and addressed in a single session to enhance understanding and monitoring of participants’ fatigue. Consequently, four offline virtual sessions were conducted weekly.

To assess the emotional requirements of individuals, a series of inquiries were employed to gauge emotional representation. The questions included: What is the primary challenge that MS has imposed on you? What are your greatest fears regarding MS? Among the primary emotional concerns identified were stress management, problem-solving, anger control, and depression management. Patients received weekly training two days following the cognitive training sessions.

#### 2.4.2 Coping

Coping strategies were integrated into the representation, complemented by two online sessions held on Friday evenings aimed at fostering optimal coping levels. In each session, two participants who exhibited significant adaptation to the disease were invited to share their positive experiences. To evaluate adaptive responses, a customized checklist based on the Cognitive Emotional Regulation Questionnaire (CERQ) was utilized. The questions included: 1. I believe I must accept the situation that has occurred. 2. I think I need to learn to coexist with this negative experience. 3. I contemplate how I can manage the situation more effectively. Following the analysis of responses, individuals demonstrating the highest levels of acceptance of the disease, with informed consent, shared their experiences with other participants. Additionally, participants were encouraged to engage with researchers during the online sessions. The researchers facilitated discussions, promoting the sharing of experiences in a manner that fostered positive coping strategies. Real-time feedback was provided, and discussions were moderated to mitigate misinformation or distressing content.

#### 2.4.3 Appraisal

To achieve this aim, researchers developed inquiries focused on both cognitive and emotional aspects. For instance: 1. What concerns you most about this illness? 2. Are you apprehensive about your recovery? After gathering feedback and evaluations, we resumed the instruction of the necessary content.

The control group did not receive any intervention during this period. Ethical considerations were upheld by offering training to the control group following the completion of the intervention.

### 2.5 Instruments

In this study, data collection comprised two components: the demographic information form and the Depression Anxiety Stress Scale (DASS) questionnaire.

#### 2.5.1 Demographic information

This form collects essential information regarding the participant and their illness, including the duration of MS, the number of relapses in the past year, and the number of hospital admissions within the same timeframe, as well as age, occupation, education level, family income, marital status, health insurance, psychosocial support, urban or rural residency, and lifestyle (independent or living with family). To ensure the validity of the content within this questionnaire, it was reviewed by ten professors at the university, and their feedback was incorporated into the final version.

#### 2.5.2 Depression Anxiety Stress Scale (DASS)

This questionnaire comprises 21 questions divided into three subscales measuring anxiety, stress, and depression, with each subscale containing 7 questions. It utilizes a 4-point Likert scale ranging from 0 (not at all) to 3 (very much), resulting in a score for each dimension that ranges from 0 to 21, where a higher score signifies more negative experiences (55). The validity and reliability of this questionnaire were assessed in Sahibi et al.’s (56) research conducted in Iran, yielding satisfactory and significant reliability and validity coefficients. Consequently, the DASS-21 meets the necessary criteria for application in psychological research and clinical settings, including studies related to multiple sclerosis (11, 57). Dependent variables were measured before, immediately after, and four weeks post-intervention in both the control and experimental groups. To comprehensively evaluate the cognitive and emotional responses of participants in accordance with Leventhal’s Model, additional tools such as the Brief Illness Perception Questionnaire (BIPQ), the Cognitive Emotion Regulation Questionnaire (CERQ), and qualitative inquiries were utilized.

### 2.6 Statistical analysis

Data analysis was performed using SPSS version 26.0, employing both descriptive statistics (means, standard deviations, frequencies, and percentages) and inferential statistics, including chi-square and Fisher’s exact tests. The Kolmogorov–Smirnov test was employed to assess data normality, confirming that the data were normally distributed. Independent t-tests and the equivalent nonparametric Mann–Whitney tests were utilized to compare quantitative measures across groups. A repeated measures analysis of variance was conducted to evaluate changes over three time points, and the Bonferroni pairwise comparison test was applied to examine temporal changes.

### 2.7 Ethics statement

In December 2023, the ethics committee of the Arak University of Medical Sciences approved this research, assigning it the reference number IR.ARAKMU.REC.1402.199. Throughout the study, the moderators adhered to ethical considerations, including informed consent, confidentiality, the right to withdraw, and compensation for any potential harm.

## 3 Results

The present study aimed to evaluate the effects of training based on Leventhal’s self-regulation model on anxiety, stress, and depression in women with multiple sclerosis, involving a total of 60 patients (31 in the control group and 29 in the experimental group). No statistically significant differences were observed between the control and experimental groups regarding age, sex, occupation, education, living situation (alone or with family), number of hospitalizations, number of relapses, urban or rural residence, insurance status, and support resources (*p* > 0.05). However, the marital status variable exhibited a significant difference between the two groups (*p* < 0.05), which was controlled in the analysis of variance with repeated measures. The average age of the patients was 37.75 ± 6.44 years, ranging from 23–50 years (Table 2).

**Table 2:** Between-group comparisons of participants’ baseline characteristics.

Group Variable			Group		*p* value
			Control (31) Mean±SD No (%)	Intervention (29) Mean±SD No (%)	
Age(years)			5.63 ± 37.87	7.31 ± 37.62	*p* = 0.882^a^
Duration(years)			6.2 ± 8.87	5.72 ± 9.83	*p* = 0.538^a^
Marital status	Divorced	(%)	1 (3.2%)	4 (13.8%)	*p* = 0.007^b^
	Single	(%)	2) 6.5%(	9) 31%(	
	Married	(%)	28) 90.3%(	16) 55.2%(	
Occupation	Employed	(%)	23 (74.2%)	20 (69%)	*p* = 0.653^c^
	Housewife	(%)	8 (25.8%)	9 (31%)	
Education	Undereducated	(%)	0	1 (3.4%)	*p* = 0.606^b^
	Under diploma	(%)	2 (6.5%)	0	
	Diploma	(%)	11 (35.5)	11 (37.9)	
	University	(%)	18 (58.1%)	17 (58.6%)	
Who life	Alone	(%)	0	2 (6.9%)	*p* = 0.229^b^
	Family	(%)	31 (100%)	27 (93.1%)	
Hospitalization How many times have you been admitted to the hospital over the last year?	Without	(%)	22 (71%)	16 (55.2%)	*p* = 0.528^c^
	Once	(%)	5 (16.1%)	7 (24.1%)	
	More than once	(%)	4 (12.9%)	6 (20.7%)	
Recurrence: How many times has your illness recurred in the past year?	Without	(%)	27 (87.1%)	24 (82.8%)	*p* = 0.576^b^
	Twice	(%)	1 (3.2%)	3 (10.3%)	
	More than twice	(%)	3 (9.7%)	2 (6.9%)	
Living: What is the location of your residence?	Village	(%)	1 (3.2%)	0	*p* = 0.999^c^
	City	(%)	30 (96.8%)	29 (100%)	
Insurance: Do you have health insurance?	Without	(%)	1 (3.2%)	1 (3.4%)	P = 0.999^b^
	With	(%)	30 (96.8%)	28 (96.6%)	
Support: What support system do you have?	No support	(%)	5 (16.1%)	0	*p* = 0.166^b^
	MS Association	(%)	8 (25.8%)	8 (27.6%)	
	Friends Support	(%)	7 (22.6%)	8 (27.6%)	
	Family support	(%)	0	0	
	all	(%)	11 (35.5%)	13 (44.8%)	

^a^Independent t test; ^b^Fisher’s exact test; ^c^Chi-square test.

Results indicated that prior to the intervention, the mean scores for depression, anxiety, and stress were not significantly different between the two groups (*p* > 0.05). However, the average scores for depression, anxiety, and stress after the intervention and one-month post-intervention were significantly different between the control and experimental groups (*p* < 0.05).

To investigate the trend of changes in stress, anxiety, and depression over three time points, a repeated measures analysis of variance was conducted, controlling for confounding effects such as marital status. The results indicated that the average changes in stress, anxiety, and depression across all samples were not significant (*p* > 0.05). However, the interaction effect between time and group was significant (*p* < 0.05), which means that there was a significant difference in time changes between the groups. In other words, the trends in the average stress, anxiety and depression in the two groups over time were significantly different (Table 3).

**Table 3:** Comparison of the mean and standard deviation changes in depression, anxiety and stress.

Variable	Groups	T1 Mean±SD	T2 Mean±SD	T3 Mean±SD	Mean±SD	Analysis of variance with repeated measures		
						Within-subject	Between-group	Time group
Stress	Intervention	5.57 ± 8.55	4.55 ± 4.93	4.38 ± 5.51	0.870 ± 6.075	*F* = 0.066 *p* = 0.803	*F* = 14.850 p = 0.0001	*F* = 6.839 *p* = 0.011
	Control	4.97 ± 11.09	5.10 ± 10.35	5.02 ± 10.41	0.840 ± 10.866			
Anxiety	Intervention	4.34 ± 4.89	2.48 ± 1.96	2.31 ± 2.93	3.054 ± 0.746	*F* = 0.717 *p* = 0.491	*F* = 19.470 *p* = 0.0001	*F* = 13.320 *P* = 0.0001
	Control	5.34 ± 7.06	4.98 ± 7.70	4.94 ± 7.90	7.756 ± 0.720			
Depression	Intervention	4.26 ± 6.82	3.73 ± 4.37	3.61 ± 4.68	0.803 ± 5.013	*F* = 2.387 *p* = 0.127	*F* = 11.769 *p* = 0.001	*F* = 10.604 *P* = 0.0001
	Control	5.33 ± 8.61	5.06 ± 8.64	4.88 ± 8.77	0.775 ± 8.945			

T1, before intervention; T2, after intervention; T3, one month after the intervention.

### 3.1 Anxiety

As presented in Table 4, the experimental group experienced a significant decrease in average anxiety scores following the intervention compared to pre-intervention levels (*p* < 0.05), but one month after the intervention, the average anxiety score increased significantly (*p* < 0.05). In the control group, there was no significant difference in average anxiety scores following the intervention (*p* > 0.05). Similar to the experimental group, the control group also exhibited a significant increase in anxiety levels one month after the intervention compared to post-intervention scores (*p* < 0.05).

**Table 4:** Results of paired comparisons of the means and standard deviations of depression, anxiety and stress over time across groups (Bonferroni *post hoc* correction).

Variable	Group	Time	Control			Intervention		
			Standard deviation	Comparison of mean	*P* value	Standard deviation	Comparison of mean	Value
Stress	Before (T1)	After (T2)	0.236	0.742	0.011	1.038	3.621	0.005
		One month after (T3)	0.243	0.677	0.027	1.065	3.034	0.024
	After intervention (T2)	One month after (T3)	0.045	0.065-	0.482	0.127	0.586-	0.0001
Anxiety	Before (T1)	After (T2)	0.414	0.645-	0.388	0.893	2.931	0.008
		One month after (T3)	0.399	0.839-	0.132	0.857	1.966	0.089
	After intervention (T2)	One month after (T3)	0.072	0.194-	0.035	0.136	0.966-	0.0001
Depression	Before (T1)	After (T2)	0.378	0.032-	0.999	0.846	2.448	0.022
		One month after (T3)	0.347	0.161-	0.999	0.855	2.138	0.056
	After intervention (T2)	One month after (T3)	0.077	0.129-	0.310	0.132	0.310-	0.079

T1, before intervention; T2, after intervention; T3, one month after the intervention.

### 3.2 Stress

The analysis revealed significant differences in average stress changes over time between the two groups. Both the experimental and control groups showed a significant decrease in average stress levels after the intervention and one-month post-intervention compared to pre-intervention levels (*p* < 0.05). However, in the experimental group, there was a significant increase in average stress levels one month after the intervention compared to post-intervention levels (p < 0.05).

### 3.3 Depression

In the experimental group, the average depression score decreased significantly after the intervention compared to pre-intervention levels (p < 0.05). At subsequent evaluation points, no significant changes were observed (*p* > 0.05). In the control group, there were no significant differences in average depression scores across the evaluation periods (p > 0.05).

## 4 Discussion

This study examined the efficacy of Leventhal’s self-regulation model in alleviating stress, anxiety, and depression among female patients with multiple sclerosis (MS). The results indicated significant enhancements in psychological outcomes immediately post-intervention; however, the sustainability of these effects differed across various psychological domains over time.

### 4.1 Anxiety

The experimental group demonstrated a significant reduction in anxiety immediately after the intervention (*p* < 0.05). However, one month later, anxiety levels significantly increased (p < 0.05), indicating that the intervention’s effect was not maintained long-term. In contrast, the control group showed no significant change in anxiety levels, except for a significant increase one month after the intervention (p < 0.05). These results suggest that while the intervention produced initial efficacy, supplementary strategies may be necessary to sustain its benefits. Previous research has shown that psychological interventions based on the illness perception framework, including the Leventhal model, can effectively reduce anxiety (58, 59). Similarly, the intergroup comparison confirmed a significant intervention effect on anxiety reduction. Specifically, no significant difference was observed before the intervention (*p* > 0.05), but after the intervention and at the one-month follow-up, the experimental group presented significantly lower anxiety levels than did the control group (*p* < 0.05). These findings align with previous research demonstrating an inverse relationship between illness perception and anxiety levels (60).

### 4.2 Depression

The experimental group showed a significant decrease in depressive symptoms after the intervention (p < 0.05), and this improvement persisted at the one-month follow-up. In contrast, the control group showed an increasing trend in depressive symptoms over time, although this change was not statistically significant. Depression is a common concern among patients with MS (61) and is often associated with the unpredictability of the disease and uncertainty about prognosis (62). Given that the intervention enhanced both the cognitive and physical aspects of illness perception, it is plausible that it contributed to a better understanding of the disease course and, consequently, a reduction in depressive symptoms. These results are consistent with previous research that emphasized the role of illness perception interventions in reducing depression in populations with chronic illnesses (63, 64).

### 4.3 Stress

Both groups presented a significant reduction in stress after the intervention (*p* < 0.05), but this decrease was not sustained over time in the experimental group. Interestingly, stress levels also decreased in the control group, suggesting that factors such as participant expectations or the study design might have influenced these outcomes. Previous research underscores that predictability and perceived autonomy significantly impact stress levels (65), a phenomenon that may explain the observed reductions in both cohorts. Nevertheless, additional longitudinal evaluations are imperative to ascertain whether the intervention yields enduring effects. Collectively, these results bolster the validity of Leventhal’s theoretical framework in enhancing psychological well-being among individuals with chronic ailments (66, 67). However, some investigations have yielded inconclusive outcomes, suggesting that supplementary behavioral or spiritual strategies may be required for sustained benefits (68). The discrepancies in the findings across these studies may be attributed to variations in the nature of the diverse diseases examined. Furthermore, differences in illness perceptions may influence other outcomes. Specifically, illness perception can affect treatment adherence and psychosocial responses, thereby impacting disease-related symptoms and outcomes such as stress, anxiety, and depression (30).

### 4.4 Conclusion

This research analyzed the effectiveness of Leventhal’s self-regulation model in reducing anxiety, stress, and depression in women diagnosed with MS. Although the intervention significantly improved psychological outcomes in the short term, its long-term sustainability remains a subject of debate. Both anxiety and stress improved immediately after the intervention but increased again after one month. The reduction in depression, however, remained stable over time.The results highlight the model’s strength in altering illness perceptions, particularly for depression, likely due to its support for cognitive restructuring and emotional adaptation. However, the temporary nature of anxiety and stress reduction points to the need for more comprehensive and sustained interventions. Despite its advantages, this intervention alone may prove inadequate for long-term psychological management in individuals with MS.

Future investigations should examine multimodal strategies, such as the integration of self-regulation training with cognitive–behavioral therapy (CBT), mindfulness-based interventions, or pharmacological treatments, to increase its overall effectiveness. Furthermore, long-term follow-up assessments are imperative to ascertain the persistence of its effects and to refine intervention methodologies conducive to sustainable mental health enhancements. Additional research should prioritize individualized intervention frameworks and the incorporation of digital or telehealth adaptations to improve accessibility and adherence.

## Data Availability

The original contributions presented in the study are included in the article/supplementary material, further inquiries can be directed to the corresponding author/s.
